# Rapid fragmentation of microplastics by the freshwater amphipod *Gammarus duebeni* (Lillj.)

**DOI:** 10.1038/s41598-020-69635-2

**Published:** 2020-07-30

**Authors:** Alicia Mateos-Cárdenas, John O’Halloran, Frank N. A. M. van Pelt, Marcel A. K. Jansen

**Affiliations:** 1 School of Biological, Earth and Environmental Sciences, University College Cork, North Mall, Cork, Ireland; 2 Environmental Research Institute, Lee Road, Cork, Ireland; 3 Department of Pharmacology and Therapeutics, Western Gateway Building, University College Cork, Western Road, Cork, Ireland

**Keywords:** Freshwater ecology, Environmental chemistry, Environmental impact, Ecology, Ecology, Environmental sciences, Zoology

## Abstract

Microplastics have become ubiquitous in all environments. Yet, their environmental fate is still largely unknown. Plastic fragmentation is a key component of plastic degradation, which is mostly caused by abiotic processes over prolonged time scales. Here, it is shown that the freshwater amphipod *Gammarus duebeni* can rapidly fragment polyethylene microplastics, resulting in the formation of differently shaped and sized plastic fragments, including nanoplastics. Fragments comprised 65.7% of all observed microplastic particles accumulated in digestive tracts. Higher numbers of fragments were found in response to longer exposure times and/or higher microplastic concentrations. Furthermore, the proportion of smaller plastic fragments was highest when food was present during the depuration process. It is concluded that *G. duebeni* can rapidly fragment polyethylene microplastics and that this is closely associated with the feeding process. These results highlight the crucial role, currently understudied, that biota may play in determining the fate of microplastics in aquatic ecosystems.

## Introduction

Microplastics are small plastic particles of 1 µm to 1 mm in size^1^ that have been reported to be ubiquitous in marine, freshwater and terrestrial environments^2,3^. In the past few years, research has revealed that freshwater habitats do not just transport plastics from land to the ocean, but are also microplastic pollution sinks^4,5^. Recent freshwater monitoring studies have reported the presence of microplastics on the water surface or in the water column^6–8^ and in sediments^9–13^. Typically the most common microplastics detected in aquatic samples are microfibres, followed by fragments and films^2^. However, freshwater studies have also reported the presence of plastic microspheres in rivers and lakes^8,10–12,14–17^.

Freshwater fish and macroinvertebrates have been found to ingest microplastics in the natural environment^18–20^. Consequently, it has been hypothesised that microplastics may have an impact on primary producer and consumer species present in aquatic environments. In fact, microplastics are now classified as freshwater contaminants of emerging concern due to their potential risks to freshwater biota and ecosystems^21^. However, rather than a generic risk, it is likely that some biota are more at risk than others. For example, factors such as feeding strategies and developmental stage may determine the uptake of microplastics and the subsequent impact on freshwater macroinvertebrates^22^. Ecotoxicological studies have shown that model aquatic organisms such as daphnids and gammarids readily ingest microplastics, possibly mistaking them for food^23–25^. For example, ingestion of 2 µm polystyrene microspheres by *Daphnia magna* was enhanced when food was absent. Yet, no effect of microplastics was detected on *D. magna* mortality or reproduction after 21 days^23^. In contrast, another study found that ingestion of 1 µm polyethylene microbeads caused *D. magna* immobilisation after 96 h^26^. Ingestion is not limited to smaller microplastics. In fact, *D. magna* is capable of ingesting 300–1,400 µm polyester microfibres^27^. In general, microfibre uptake by *D. magna* increased with higher microplastic doses and only caused mortality after 48 h when daphnids had not been pre-fed^27^. Other ecotoxicological studies have shown that the model species *Gammarus *sp*.* can also ingest microplastics. It was shown that 10–45 μm polyethylene microplastics are bioavailable to the freshwater amphipod *Gammarus duebeni* when these plastics are adsorbed to plant material^25^. In this study, *G. duebeni* contained low numbers of microplastics in the gut and no mortality was observed after 48 h. Another study showed that *Gammarus pulex* body microplastic burden was dose-dependent in terms of microplastic concentration when fed polyethylene terephthalate fragments for 24 h^28^. However, no effect was found on feeding, energy reserves, moulting or mortality after 48 d^28^. While these studies abundantly show that ingestion of microplastics by invertebrates does occur, the fate of plastics in regard to fragmentation inside organisms after ingestion is still largely unknown.

Future scenarios suggest an increment of plastic pollution entering natural environments as plastic production increase in business as usual scenarios^29,30^. Also, it is estimated that 99% of the global plastic waste entering the oceans goes ‘missing’, pointing towards gaps in knowledge regarding microplastic fate^31^. The lack of knowledge of the environmental fate of plastics is therefore a major issue. A recent paper indicated one possible fate of ingested microplastics in Antarctic krill, that is digestive fragmentation of microplastics into nanoplastics^32^. Another study showed the damage to large pieces of plastics such as expanded polystyrene buoys (EPS) by wild polychaetes collected living on these buoys. The same study also showed that polychaetes can produce EPS fragments of 1–5 mm during burrowing under laboratory conditions^33^. Yet, the concept of biological fragmentation of plastics is underexplored and it remains to be shown whether this is a process unique to a small number of species, or whether it is more widespread throughout the natural world. The importance of digestive fragmentation relates to the environmental fate of microplastics, and the potential generation of large numbers of nanoplastics with substantially unknown impacts^34^. Our preliminary observations in the current study indicated potential accumulation of plastic fragments in *G. duebeni* digestive tracts after short polyethylene microplastic feeding tests. We hypothesised that the freshwater crustaceae *G. duebeni* has the ability to fragment microplastics. This hypothesis was tested by studying microplastic ingestion and fragmentation using different microplastic concentrations, exposure times and depuration types. *G. duebeni* was selected for microplastic studies as a representative of the amphipods, small crustaceans from the order Amphipoda, that are keystone and model ecotoxicological species^35^ which are widespread in marine and freshwater global environments. Our findings of this study show that biological fragmentation of microplastics may have an important role in determining the fate of plastics in the environment worldwide.

## Results

### Exposure time and microplastic concentration co-determine microplastic accumulation and fragmentation by *Gammarus duebeni*

*G. duebeni* were individually exposed to microplastics in the absence of food. The experimental design comprised three variables (1) two microplastic concentrations; low or high, (2) two different exposure times; 24 h or 96 h and (3) three depuration types after microplastic exposure; no depuration, a 24 h depuration in presence of food or a 24 h depuration in absence of food (Figure S1). From a total of 108 *G. duebeni* adults, 72 were exposed to 10–45 µm spherical polyethylene microplastics (MPs) while 36 belonged to the non-exposed control groups. Survival was monitored. Overall, 104 individuals survived the experiment (3.7% test mortality). The four *G. duebeni* which died belonged to the following treatment groups: (I) 24 h, low microplastic concentration and depuration in presence of food, (II) 96 h, low microplastic concentration and no depuration, (III) 96 h, low microplastic concentration and depuration in presence of food and (IV) 96 h, high microplastic concentration and no depuration. None of the control *G. duebeni* individuals were found to contain microplastics, or microplastic fragments. Visual observations showed that amphipods did not produce faecal pellets during microplastic exposure or the subsequent depuration period. Further microscopy observations of the filtered water column confirmed the absence of faecal pellets or fragments.

A total of 34 amphipods, from the 72 that were exposed to microplastics, contained microplastics. For the purpose of quantifying microplastic occurrence in *G. duebeni*, the number of intact microplastics, as well as fragments, were counted in digestive tracts. Microplastic ingestion per sé cannot be accurately quantified due to fragmentation. Each ingested microsphere is likely to produce more than one fragment, and therefore this study refers to accumulation of microplastics.

**Figure 1:**
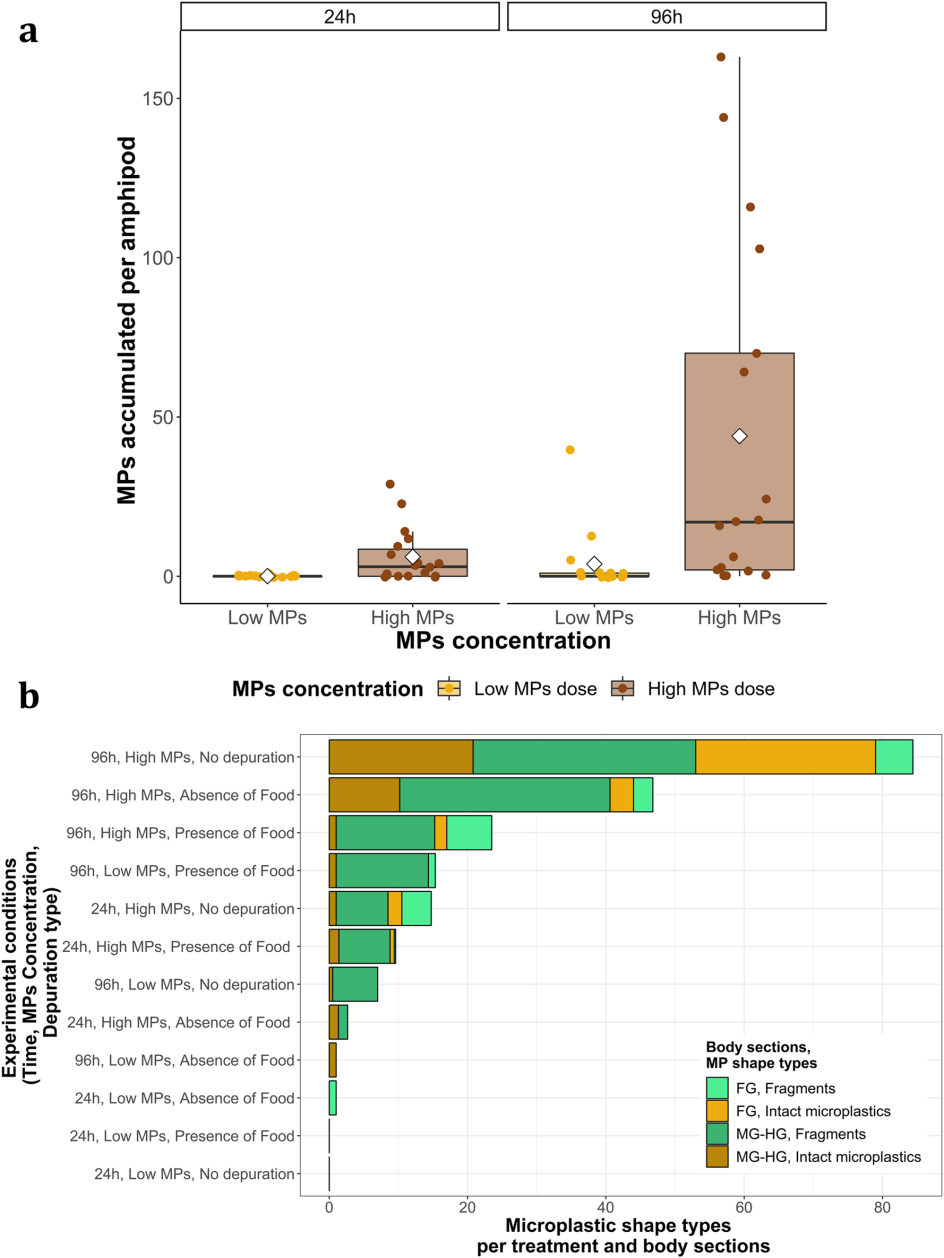
Microplastic accumulation in *G. duebeni* digestive tracts under experimental conditions such as: time (24 h or 96 h exposure to plastics), microplastic concentration (Low MPs or High MPs) and depuration type (no depuration, 24 h depuration in presence or absence of food). Six replicates were run. All amphipods were exposed individually. (**a**) Shows the number of microplastics accumulated in *G. duebeni* for 24 and 96 h and for the two microplastic concentrations tested: low (600 microplastics/mL) or high 60,000 microplastics/mL). Here scatter data points represent each individual amphipod that had been exposed to microplastics (a total of 72) with its corresponding number of microplastics accumulated, including those amphipods with zero microplastics. Boxplots midline represents the median. White diamonds show the mean. Lower and higher limits of the boxes represent first Q1 and third Q3 quartiles (25th and 75th percentile). The upper whisker represents Q3 + (1.5 × IQR). IQR is the interquartile range. (**b**) Shows the average number of microplastics for each body section (“FG” stands for “Foregut” and “MG-HG” for “Midgut and Hindgut”) and microplastic shape type (intact microplastics or fragments). Here data is shown for all amphipods within each treatment, including those amphipods that had not accumulated particles. Both figures were produced using the “ggplot2” package in R (v3.4.3).

Microplastic occurrence in *G. duebeni* was highly dependent on experimental conditions, including microplastic exposure time, microplastic concentration and depuration type (MANOVA, F = 6.63, df = 1, p value < 0.001). Microplastic concentration (p value < 0.001) and exposure time (p value < 0.01) significantly contributed to the number of microplastics accumulated in amphipods (Fig. 1a). *G. duebeni* accumulated varied quantities of microplastics (both intact microspheres and fragments) depending on the time and/or microplastic dose they had been exposed to (Fig. 1a,b). In summary, (I) only one amphipod accumulated 1 microplastic after 24 h exposure to the low microplastic dose, (II) twelve amphipods accumulated an average of 9.2 ± 2.6 microplastics (mean ± SE) after 24 h exposure to the high microplastic dose, (III) seven amphipods accumulated an average of 8.9 ± 5.5 microplastics after 96 h exposure to the low microplastic dose and (IV) fourteen amphipods accumulated an average of 53.4 ± 15.2 microplastics after 96 h exposure to the high microplastic dose (Fig. 1a).

The number of microplastic particles present in midgut and hindgut sections was higher with increasing exposure time and microplastic concentration (Fig. 1b; Figure S2). A general trend can be observed in Fig. 1b, more microplastics are accumulated after a longer exposure time to a higher MPs dose (top three stacked bars) compared to very low numbers of, or even zero, microplastics that were accumulated after a shorter time exposure to the lower MPs dose (bottom three stacked bars). To further investigate the effects of microplastic concentration and exposure time, the amphipods grouped under “no depuration” were selected for an additional statistical analysis. A significant interactive effect between microplastic concentration and exposure time was noted (Two-way ANOVA, F = 7.91, df = 1, p value < 0.05). A multiple comparison of means Tukey post-hoc test showed microplastic occurrence in amphipods to be significantly enhanced under the following treatments (1) exposure to high microplastic concentration during 96 h compared to during 24 h (p value < 0.01) as well as (2) exposure to high microplastic concentration during 96 h compared to exposure to low microplastic concentration during 24 h (p value < 0.01) and (3) exposure to high microplastic concentration compared to low microplastic concentration both during 96 h (p value < 0.01). The number of microplastics accumulated by *G. duebeni* in the absence of depuration was significantly greater when organisms were fed the higher dose of microplastics (Two-way ANOVA, F = 10.67, df = 1, p value < 0.001) and when they were exposed for a longer time (96 h) to microplastics (Two-way ANOVA, F = 9.18, df = 1, p value < 0.01). The presence or absence of a 24 h depuration period had an effect on the total number of microplastics found in amphipod digestive tracts (Fig. 1b, Tables S1 and S2). Overall, there was a higher number of microplastics in amphipods exposed to microplastics in the absence of depuration (a total number of 489 microplastics across all amphipods in this treatment, 181 were in the foregut and 308 in the midgut and hindgut, Table S2), compared to amphipods that had a 24 h depuration time. Likewise, the number of microplastics in the amphipods was higher after a 24 h depuration in the absence of food (a total of 317 microplastics across all amphipods, of which 40 were in foregut and 277 in midgut and hindgut, Table S2) when compared to a 24 h depuration in presence of food (a total of 188 microplastics, of which 40 were in foregut and 148 in midgut and hindgut, Table S2).

**Figure 2:**
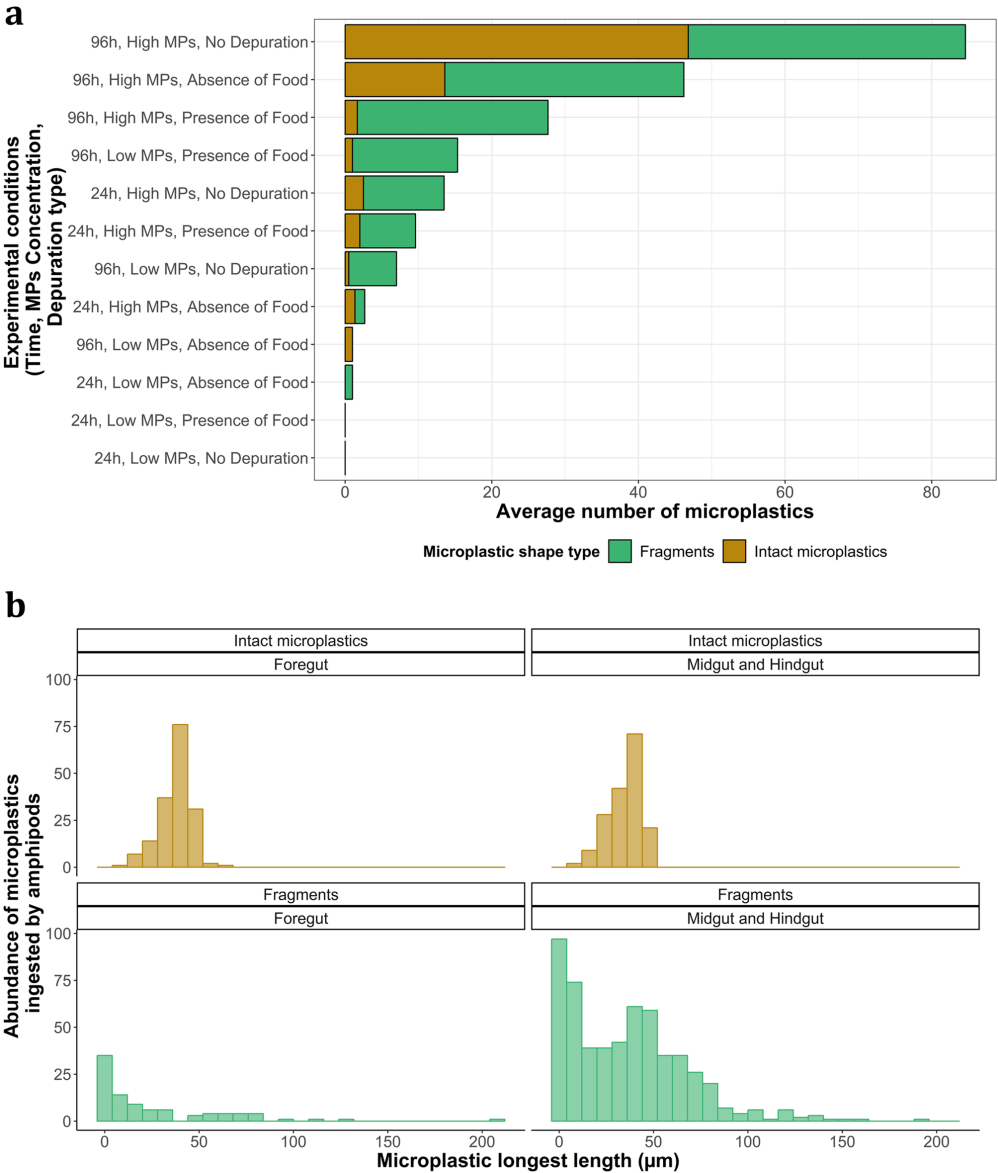
Microplastic fragmentation by *G. duebeni.* Six replicates were run. All amphipods were exposed individually. (**a**) Shows average number of microplastics present in all *G. duebeni* according to their shape types (intact microplastics or fragments) per treatments. The presence of intact microplastics and plastic fragments in *G. duebeni* varied as a function of different experimental treatments such as: time, microplastic concentration and depuration type. Data is shown per treatment. (**b**) Shows the abundance of intact microplastics and plastic fragments of different size ranges accumulated in all *G. duebeni* foreguts and midguts-hindguts. Both figures were produced using the “ggplot2” package in R (v3.4.3).

Of a total of 994 microplastics found in all *G. duebeni*, 653 were plastic fragments, which comprised 65.7% of all microplastics found (Fig. 2a, Table S2). The number of microplastics, particularly fragments, found in *G. duebeni* midgut-hindgut sections was significantly higher compared to that in foregut sections (Figs. 1b, 2a; Figure S2, MANOVA, F = 4.21, df = 1, p value < 0.05). The number of microplastic fragments increased with increasing microplastic exposure time and concentration. The exception was the 96 h exposure treatment to a high microplastic concentration in the absence of depuration, where a slightly higher number of intact microspheres was noted instead (Figs. 1b, 2a). There was an effect of depuration on the ratio of fragments to whole microplastics within *G. duebeni* individuals. This ratio (fragment:intact) was 1:1 in the absence of depuration, 3:1 for depuration in the absence of food and 7:1 for depuration in the presence of food.

### Microplastics are fragmented in a variety of shapes and sizes

A variety of plastic fragment sizes was found in *G. duebeni* across treatments (Fig. 2b). The highest number of fragments was found in *G. duebeni* midgut and hindgut sections, with an average size of 36.22 ± 1.31 µm (mean ± SE). Nanoplastics were also present in midgut and hindgut sections with an average size of 0.76 ± 0.13 µm. Fragments found in foregut sections had an average size of 25.52 ± 3.65 µm. Nanoplastics were also present in foregut sections with an average size of 0.68 ± 0.07 µm. Intact microplastics found in the foregut section and the combined midgut and hindgut sections had an average size of 37.43 ± 0.67 µm and 35.35 ± 0.65 µm, respectively.

**Figure 3:**
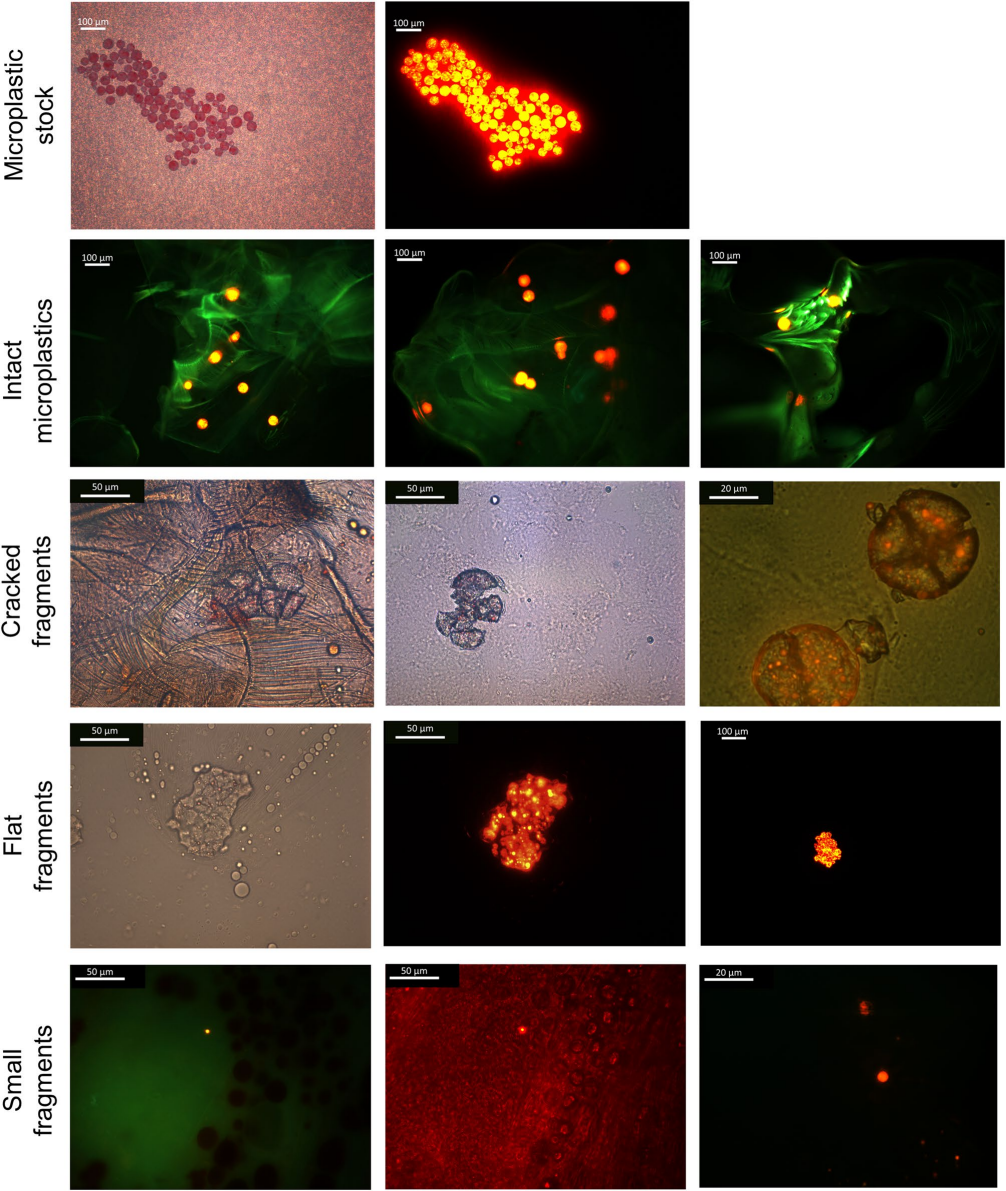
Fluorescence and light microscope images of intact microplastics and plastic fragments found in *G. duebeni* digestive tracts.

A detailed analysis of fragmentation using bright field and fluorescence microscopy showed fragmented microplastics of varied morphologies and sizes (Fig. 3; Figure S3). Fragmented microplastic shapes were described as ‘small irregular’, ‘flat’ or ‘cracked (semi-spherical in shape)’ fragments. The most common fragments found in *G. duebeni* were cracked (301), followed by small irregular (236) and flat (116) plastics. Intact microplastics had a spherical form and ranged in size between 10–45 µm diameter.

**Figure 4:**
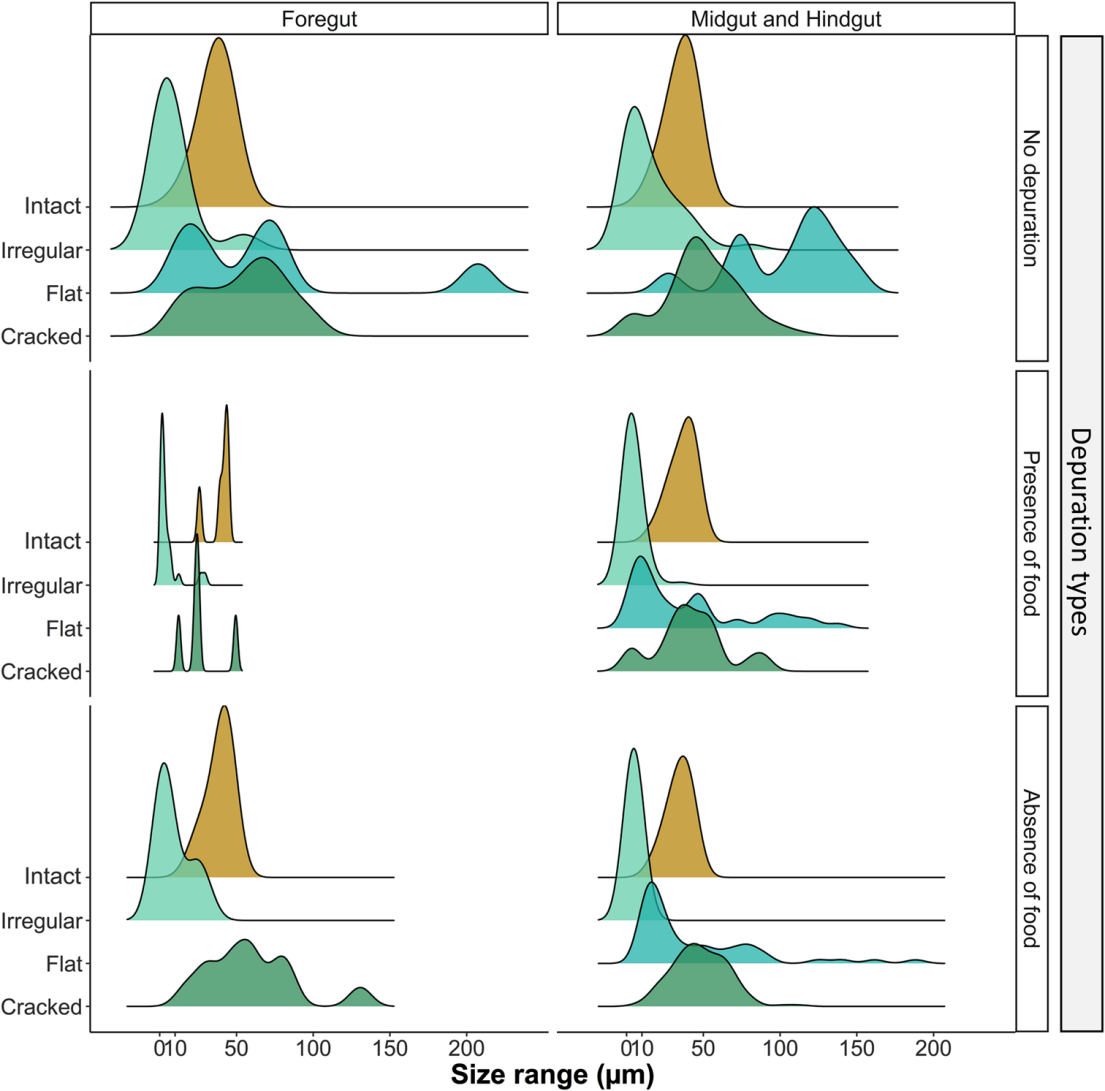
Size distribution of all microplastic shape types found in all *G. duebeni* foregut (head), midgut and hindgut (thorax and abdomen) sections according to depuration types. The height of the ridgelines shows the sum of microplastics of different sizes and shapes. Six replicates were run. All amphipods were exposed individually. This figure was produced using the packages “ggplot2” and “ggridges” in R (v3.4.3).

A total of 994 microplastics, including both intact microspheres and fragments, were found in *G. duebeni* across all treatments. Overall, more microplastic fragments (653) were found in *G. duebeni* than intact microspheres (341). The most common fragment type found in *G. duebeni* were cracked fragments (301), followed by small irregular fragments (236) and flat fragments (116). A range of microplastic fragment sizes was found in *G. duebeni* across microplastic treatments. Fragment sizes ranged from nanoplastic fragments (558 nm–1 µm in length) to microplastic fragments, some of them being larger in size than those from the original stock (longest microplastic fragment found was 207.3 µm). Overall, the size distribution (0.5–250 µm) of all microplastics found in *G. duebeni* (Fig. 4) was significantly different depending on depuration type (MANOVA, F = 25.22, df = 1, p value < 0.0001). Depuration type also had a significant effect on the size of microplastics present in foreguts (Fig. 4, MANOVA, F = 27.97, df = 1, p value < 0.0001, Tables S2 and S3) and midgut and hindgut sections (Fig. 4, MANOVA, F = 31.82, df = 1, p value < 0.0001), Tables S2 and S3). The smallest plastic fragments were found in foreguts (5.03 ± 2.69 µm) and midguts–hindguts (4.19 ± 2.49 µm), when food was present during depuration.

### Quality control of plastic particle sizes and fluorescence dye

To ascertain the role of *G. duebeni* in microplastic fragmentation, quality control experiments were performed and these included mock-treatments to simulate all experimental stages and steps (i.e. pristine microplastics as powder, suspended microplastics in Tween, and microplastics after 7 days in − 80 °C freezer), but in the absence of amphipods. Scanning electron microscopy (SEM) images of microplastic stock (Figure S4) showed that microplastics shape and size remained intact at every step of the experimental handling process. SEM images also showed that the microplastic stock did not contain micro- or nanoplastics outside the size range of 10–45 µm. Microplastics were mounted on slides using the same procedure at all times, and no fragmentation was observed for those microplastics that had not been in contact with an amphipod.

All fluorophores accumulating within internal *G. duebeni* tissues were found to be associated with a microplastic particle. Results from images of *G. duebeni* exoskeleton or gut tissues (Figure S5), showed biological autofluorescence of control individuals, however this was distinct from the fluorescence by the microplastic fluorophore. This clearly demonstrated that *G. duebeni*’s biological autofluorescence did not interfere in the accurate detection of detected plastic particles (which are shown in Fig. 3 and Figure S3).

## Discussion

Microbeads of different polymers, including PE, are commonly present in freshwater samples. Particularly, urban rivers may be microbead hotspots^12^. For example, spherules made up 60% of the microplastics found in the river Rhine^8^ while also being found in many other river systems such as St. Lawrence River sediments^9^, and both sediment and surface water of the Ottawa River^10^. Amphipods have been reported to ingest microplastics possibly by mistaking them for food^36^. In this study, *G. duebeni* contained microplastics, both fragmented and intact. Microplastic accumulation appeared particularly enhanced when amphipods were exposed to a higher microplastic concentration and a longer exposure time. In our study, the highest number of microplastics that amphipods accumulated was 53.4 ± 15.2 microplastics/individual with no substantial mortality. This is consistent with results from previous studies which observed no mortality after different amounts of microplastics had been ingested by amphipods. For example, no mortality was found after limited uptake (1–2 microplastics/amphipod) of 8 µm PS microbeads by *Echinogammarus marinus*^24^^,^ 10–45 µm PE microbeads by *G. duebeni*^25^ and 20–500 µm PS fragments by *G. pulex*^37^. Likewise, no mortality was found after uptake of an average of 10 microplastics/amphipod of 500 × 20 µm PA microfibers, or 32–250 µm biodegradable and acrylic fragments by *Gammarus fossarum*^38,39^. Remarkably, no mortality was found in *G. pulex* that had ingested up to several thousand 150 µm PET fragments^28^.

Here we found that the amphipod *G. duebeni* can effectively fragment 10–45 µm microplastics into a range of sizes including nanoplastics (558 nm–1 µm). Plastic fragments, derived from red-fluorescent plastic microspheres, were identified inside *G. duebeni* by using fluorescence microscopy followed by brightfield microscopy at different magnifications as an additional particle verification step. This microscopy combination method was used to avoid overestimation of potential dye leachates or other artefacts as plastic fragments^40–42^. To ascertain the role of *G. duebeni* in microplastic fragmentation, control microplastic experiments were run at all experimental or handling steps but in the absence of the amphipod (i.e. microplastics from stocks and in Tween suspension, microplastics after 7 days in − 80 °C freezer, and microplastics mounted onto slides for microscopy). No fragments were observed in such mock experiments, which supports the conclusion that microplastic fragmentation happened as a biological process mediated by *G. duebeni*.

In the literature, plastic fragmentation has largely been attributed to physicochemical processes^43,44^. This includes processes such as UV photodegradation^44–46^, a combination of UV degradation and mechanical abrasion^47^ and a synergistic effect of oxidative degradation and microbiological activity^48^. A study by Song et al. (2017) concluded that abiotic processes such as UV photooxidation combined with mechanical abrasion cause plastics to become brittle and fragment after a prolonged period of time. Such embrittlement varied according to polymer type. Polyethylene was the polymer that produced the least number of fragments compared to polypropylene and styrofoam under the same experimental conditions. The study by Song et al. (2017) also estimated that plastics in a beach environment would need more than 4.2 years to fragment. Another study on plastic weathering hypothesised that the abiotic fragmentation of plastics in the freshwater environment would happen even slower than in the marine environment^49^. In contrast, our finding of microplastic fragmentation by a freshwater amphipod can be observed after short periods of time (i.e. max. 96 h). This is extremely fast in comparison with the aforementioned abiotic processes that are currently thought to drive plastic fragmentation. This difference in timescale highlights the environmental relevance of the observed digestive fragmentation in the context of the overall fate of plastics in the environment.

Freshwater amphipods rapidly fragmented microplastics into a wide range of sizes, including nano fragments. Biological fragmentation from microplastics to nanoplastics, has previously only been reported for Antarctic Krill *Euphausia superba*^32^. However, three more studies have shown other forms of biological metabolism of larger macroplastics. It was reported that 5 mm expanded polystyrene (EPS) particles, with an appearance similar to that of the original buoy material, were visible inside wild polychaetes *Marphysa sanguinea* collected living on such EPS buoys^33^. Also, a laboratory study with urchins, *Paracentrotus lividus,* attached to a polyethylene tray, showed the presence of microplastics ranging in size between 118 µm–15.8 mm^50^. Lastly, a recent monitoring study claimed that langoustines collected from the deep-sea had retained and fragmented plastics in their guts^51^. These studies suggest that digestive fragmentation can potentially play a critical role in determining the fate of plastics in the environment.

In our study, we found that increasing the exposure time and microplastic concentration led to both increased microplastic accumulation and fragmentation in *G. duebeni*. Contrary to this finding, Dawson et al. (2018) observed that plastic fragmentation by Antarctic krill was inhibited by a repeated exposure to high microplastic doses. In the present study, all amphipods that had ingested microplastics showed fragmentation. However, there was a higher ratio of fragments over intact microplastics in those amphipods which had undergone depuration in presence of food. Thus, *G. duebeni* were more efficient in fragmenting microplastics when food was present during the depuration. Likewise, the presence of food during depuration also had an effect on plastic fragment shape and size. Small irregular plastic fragments, including some in the nano scale, were the most common fragments present in *G. duebeni* foreguts and midgut/hindguts after food depuration. This indicates that food supply is a key factor that can stimulate the biological fragmentation of plastics. In fact, it was suggested that the presence of sharp edged algae and silica diatoms in the diet of *E. superba* was a potential fragmentation mechanism^32^.

More plastic fragments were found in midgut and hindgut (thorax and abdomen) sections compared to foregut (head) sections. However, the finding of fragments in foregut sections suggests that plastic fragmentation can happen early in the digestive process. Gammarids (G*ammarus *spp.) are omnivores with occasional predation and cannibalism behaviour. The functional feeding group of freshwater amphipods is leaf-shredding detritivores^35,52,53^. The feeding appendages and alimentary canal of Gammarids have been studied in detail^54–58^. *Gammarus sp.* use their antennae and gnatophods to capture food which then passes to the mandibular palps^57^. Amphipod mandibles are paired with toothed incisors, molars, bristles and a setal row for masticating and grinding food^55^. It is the articulated mandible that enables amphipods to shear off, stretch out and flatten pieces of food, especially prey tissues^56^. After the foregut, food is triturated and then passes into the midgut and a ventral chamber that acts as a filter apparatus before passing to the hindgut or rectum^54^. The pH across the alimentary canal of amphipods is only very slightly acidic (pH 6.5–6.8)^54^. Digestive enzymes, such as amylase, cellulose, esterase, protease and lipase are present in the midgut. It can be speculated that plastic fragmentation is associated with exposure to mechanical forces, gut enzymatic processes or a combination of the two. Furthermore, the intestinal microbiome has also been suggested to play a role in the degradation of polyethylene^59^.

Our observations detailed in this study reveal the potential of a group of widespread freshwater and marine species to rapidly fragment microplastics and, consequently, increase the number of irregular plastic fragments of different sizes. Furthermore, the finding that species such as *G. duebeni* can also produce numbers of nanoplastics suggests a new pathway for the environmental presence of nanosized plastics, which are of high concern as they can potentially pass through cell wall barriers and produce adverse effects. Adverse effects of nanoplastics have already been reported in microalgae^60–62^, aquatic^63^ and terrestrial^64,65^ plants, daphnids^60,66^ or blue mussel larvae^67^. Moreover, the findings presented here can also be highly relevant for plastic modelling studies as biological fragmentation is currently not considered in the fate of plastics in the environment^68^. The capacity to rapidly produce plastic fragments through digestive processes needs to be furtherly analysed as a potential determinant of the unknown fate and impacts of plastics in the aquatic environment.

## Methods

### Microplastic stock suspension

Polyethylene microplastics that had been stained fluorescent red, were provided in dry hydrophobic powder form by Cospheric (Santa Barbara, CA, USA; Product reference UVPMS-BR-0.995). Fluorescent dye is incorporated into the polymer matrix and it is therefore located inside the plastic microbeads. Additional characteristics of the microplastics tested in this study were a spherical shape, diameter of 10–45 μm, a density of 0.985 g/cm^3^ and a peak of fluorescence at 605 nm. A 20% w/v stock solution was prepared following the same procedure as^25^. The actual mean microplastic diameter was verified on ImageJ by measuring N = 20 microplastics randomly selected from Figure S4a. The exact mean diameter ± SE was calculated as 30.28 ± 2.78 µm.

Polyethylene was selected for this study because it is one of the most common polymers found in personal care products and, consequently, in aquatic systems^21,69,70^. With a density lower than water, PE microplastics tend to float, however this did not interfere with the ability of freely moving amphipods to interact with and ingest these microplastics. *G. duebeni* are able to collect food from the water surface, including floating duckweed^25^.

### Test organism

Our test organism, the freshwater species *G. duebeni*, is commonly found living in the benthos of streams and rivers in southern Ireland and England. Populations of the freshwater amphipod *G. duebeni* were collected in June (replicates 1–3) and October 2019 (replicates 4–6) from a local stream in Co. Cork, Ireland (Coordinates 51°55′07.0″N 8°37′46.5″W). Amphipods were collected using the kick-sampling technique and transported in bags filled with stream water. Immediately after collection, amphipods were transferred to 5 L tanks filled with stream water for an acclimatisation period of 48 h.

### Exposure design

A total of six replicates, involving a total of 108 *G. duebeni* individuals of which 36 were control and 72 microplastic treated, were individually starved in 100 mL beakers filled with 100 mL aerated and dechlorinated tap water for 24 h prior the start of the experiment. This step was undertaken to allow amphipod gut clearance. Observation of the presence of faecal pellets happened after 6 h during the preincubation period only. After this gut clearance step, all amphipods had egested faecal pellets during this stage prior the microplastic feeding studies. This would be expected after amphipods had been collected in the wild and kept in an acclimatisation tank with stream leaves. Each *G. duebeni* was individually exposed to either a low concentration of 600 microplastics/mL or a high concentration of 60,000 microplastics/mL for a short time exposure of 24 h or a longer time exposure of 96 h. Amphipods were not fed during the microplastic exposure time. After exposure to microplastics, each *G. duebeni* was transferred to a clean 100 mL beaker filled with 100 mL aerated and dechlorinated tap water for a 24 h depuration period that consisted on depuration in presence or absence of food (Figure S1). A number of water samples were collected for microscopy observation: a filtered water column sample after microplastic exposure, and a filtered water column sample after the depuration phase. The water column from the “contaminated” microplastic exposure beaker and the water column from the “clean” depuration beaker were individually poured into a borosilicate glass filtration unit (reference code FUC3-1K0-001, Labbox Ireland) and filtered using Isopore Membrane Filters. A pore size of 0.2 µm was selected to ensure any potential nanofragments were captured. Characteristics of the filters were hydrophilic polycarbonate membrane, 0.2 µm pore size, 47 mm diameter (reference code GTTP0470, Sigma-Aldrich Ireland). Once water samples were filtered, filters were individually labelled and separately stored in 55 mm diameter petri dishes in the − 80 °C freezer for microscopic analysis. All amphipods were individually transferred to clean distilled water and allowed to swim freely for 20 s. After this, *G. duebeni* were frozen and kept at − 80 °C prior dissection. A third no depuration period treatment was also run, meaning that *G. duebeni* individuals were frozen straight away after 24 h or 96 h exposure to microplastics. Amphipods did not produce faecal pellets during microplastic exposure or the subsequent depuration period. Further microscopy observations of the filtered water column confirmed this.

### *G. duebeni* digestive tract dissections

Prior dissections, amphipods were washed with distilled water and checked under a dissection microscope for microplastics on the exoskeleton. Foregut and Midgut-Hindgut sections of *G. duebeni* digestive tracts were dissected for microplastic inspection^71,72^. Each gut section was mounted on clear glass microscope slides (25.4 × 76.2 mm, 1–1.2 mm thick) and covered with cover glass (18 × 18 mm, 0.13–0.17 mm thick) and labelled for microscopy analyses. Dissection slides were stored in a cold room until microscopy. Microscopy analyses were carried out less than 48 h after dissections to avoid the appearance of and potential disturbance by microorganisms on the quality of images and fragmentation results.

### Fluorescence and light microscopy

A combination of fluorescence microscopy and brightfield microscopy was used to avoid misidentification of microplastics. Digestive tracts were first scanned under green fluorescence light, and detected microplastics (intact and fragments) were additionally visualised thoroughly under brightfield to verify their identity (Fig. 3 and Figure S3). All samples were visualised under a Leica DFC490 fluorescence microscope. Stock microplastics have a fluorescence emission peak of 605 nm when excited at 575 nm. Plastic particles were detected under Green light (Filter cube N2.1, Excitation filter BP 515–560) and UV/violet light (Filter cube D, Excitation filter BP 355–425) at 10 ×, 40 × or 100 × magnification. A total of 262 fluorescence images were taken and 994 microplastics were counted. For this study, the number of microbeads and/or fragments in the size range between 550 nm and 210 µm were quantified. Microplastic longest length was measured using the software ImageJ. Supplementary Figures S6a–d show how plastic particles were measured in this study. It is not evident why cracked and flat microplastics can be relatively elongated. It can be speculated that fragments, and especially flat fragments, are squeezed during fragmentation resulting in more elongated structures. No fragments smaller than 0.5 μm were detected. We acknowledge that a potential limitation of the current set up and fluorescence microscopy approach is that it does not allow observation of smaller nanoplastics.

### Microplastics quality control under scanning electron microscopy (SEM)

Scanning electron microscopy (JEOL JSM-IT100) was used to generate images of the microplastic stock. Furthermore, microplastics were subjected to various treatments to explore fragmentation due to handling of plastics. These treatments included microplastic stocks in powder form and in Tween suspension as well as microplastics stored in a − 80 °C freezer for 7 days. This quality control was designed to investigate whether (1) fragmentation was taking place as a result of the handling of microplastics or (2) microplastic stocks were already contaminated with plastic fragments in the nano size range.

### Statistical analysis

A two-way analysis of variance (Two-way ANOVA) was conducted on the influence of the three tested variables (time, microplastic concentration and depuration type) on the number of microplastics ingested by amphipods. Test time consisted of two levels (24 h and 96 h), microplastic concentration had two levels (low and high) and depuration type consisted of three levels (no depuration, depuration in presence of food and depuration in absence of food). Furthermore, a MANOVA was conducted to analyse plastic fragmentation with the additional variables of plastic shape (intact microplastics or fragments) or body location (foregut or midgut and hindgut). Statistical analysis was conducted in R (version 3.4.3). Figures were produced using the packages ggplot2 and ggridges. Other R packages used were forcats, ggjoy and tidyverse.

## Supplementary information

Supplementary information.
