# Supplementary material for: Rapid fragmentation of microplastics by the freshwater amphipod Gammarus duebeni (Lillj.)
Source: Sci Rep. 2020 Jul 30;10:12799. doi: 10.1038/s41598-020-69635-2 (PMC7393071; doi:10.1038/s41598-020-69635-2)
Supplement: Supplementary file 1 — Supplementary information. [file 41598_2020_69635_MOESM1_ESM.pdf]

Supplementary material

Rapid fragmentation of microplastics by the freshwater amphipod

Gammarus duebeni

Alicia Mateos-Cárdenas *et al.*

(Corresponding author: alicia.mateoscardenas@ucc.ie)

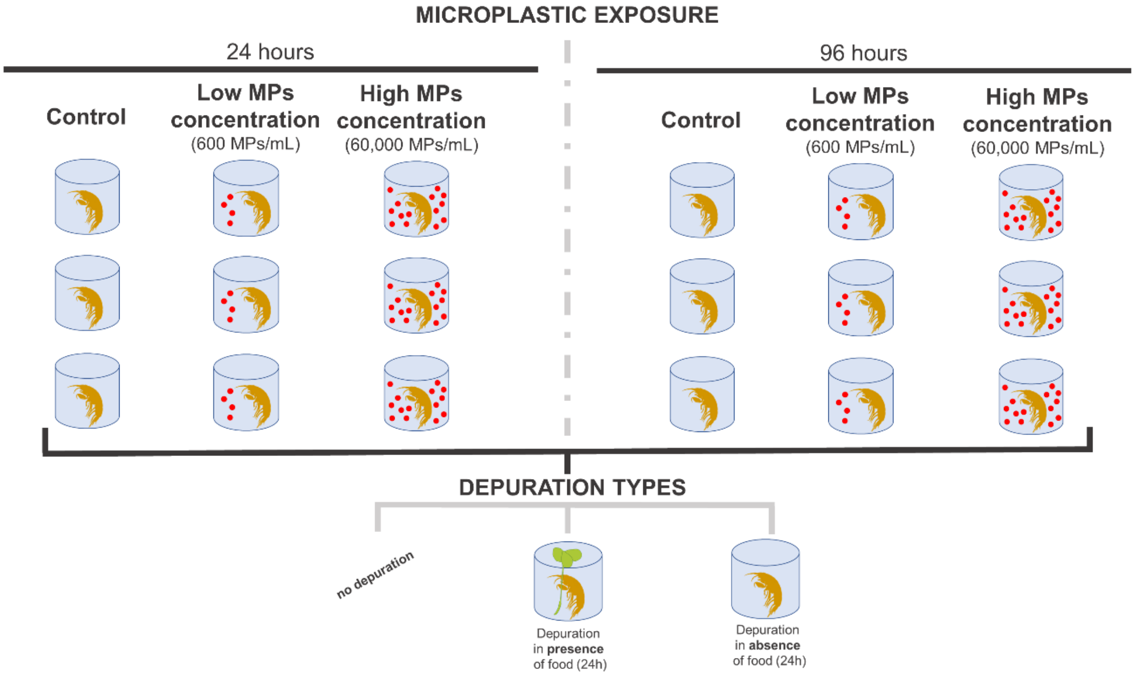

Supplementary Figure S1. Experimental design showing a graphic overview of the setup for one replicate. Six replicates were run. All amphipods were exposed individually. The experiment involved a total of 108 amphipods exposed among two microplastic doses, two different time exposures and three depuration periods. There were N = 36 control individuals (microplastic free), N = 36 individuals exposed to the low microplastic concentration and N = 36 individuals exposed to the high microplastic concentration. So, at the end of the test, a total of six *G. duebeni* had been individually exposed to different treatments consisting in: MPs dose/time/depuration type.

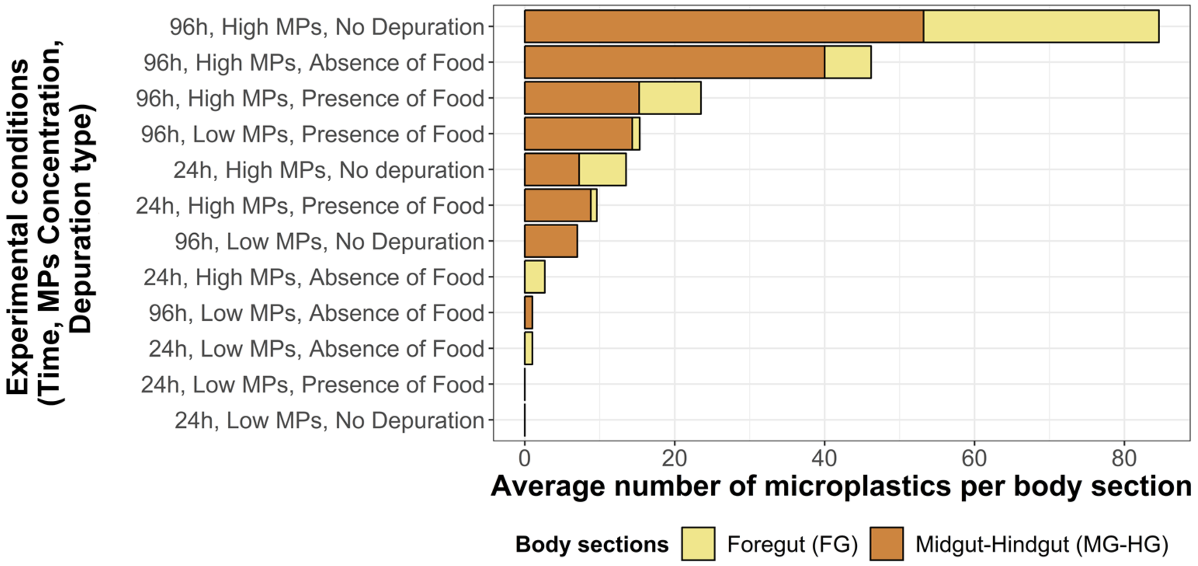

Supplementary Figure S2. Average number of microplastics present in *G. duebeni* digestive tract sections, and as a function of different experimental treatments such as: time (24h or 96h exposure to plastics), microplastic concentration (Low MPs or High MPs) and depuration type (No depuration, 24h depuration in Presence or Absence of Food). The figure shows the average number of microplastics for each body section ("FG" stands for "Foregut" and "MG-HG" for "Midgut and Hindgut"). Here data is shown for all amphipods within each treatment. This figure was produced using the "ggplot2" package in R (v3.4.3).

Supplementary Table S1. Detailed information on the microplastic (MP) presence and numbers of microplastics per *G. duebeni* individual sample according to microplastic dose, time exposure and depuration type. Control individuals (microplastic free) are not shown. Median length (µm) is given as well as mean ± SE length (µm).

Replicate	Sample ID	MPs dose	Time	Depuration	MP presence in amphipod	Total MPs	Median Length (µm)	Mean Length (µm)	SE (µm)
1	MP 01	Low MPs	24h	No depuration	no	0	X	X	X
2	MP 02	Low MPs	24h	No depuration	no	0	X	X	X
3	MP 03	Low MPs	24h	No depuration	no	0	X	X	X
4	MP 37	Low MPs	24h	No depuration	no	0	X	X	X
5	MP 38	Low MPs	24h	No depuration	no	0	X	X	X
6	MP 39	Low MPs	24h	No depuration	no	0	X	X	X
1	MP 04	Low MPs	24h	Presence of food	no	0	X	X	X
2	MP 05	Low MPs	24h	Presence of food	no	0	X	X	X
3	MP 06	Low MPs	24h	Presence of food	no	0	X	X	X
4	MP 40	Low MPs	24h	Presence of food	no	0	X	X	X
5	MP 41	Low MPs	24h	Presence of food	no	0	X	X	X
6	MP 42	Low MPs	24h	Presence of food	mortality	X	X	X	X
1	MP 07	Low MPs	24h	Absence of food	no	0	X	X	X
2	MP 08	Low MPs	24h	Absence of food	no	0	X	X	X
3	MP 09	Low MPs	24h	Absence of food	no	0	X	X	X
4	MP 43	Low MPs	24h	Absence of food	yes	1	1.58	1.58	0.00
5	MP 44	Low MPs	24h	Absence of food	no	0	X	X	X
6	MP 45	Low MPs	24h	Absence of food	no	0	X	X	X
1	MP 20	Low MPs	96h	No depuration	yes	1	37.83	37.83	0.00
2	MP 21	Low MPs	96h	No depuration	mortality	X	X	X	X
3	MP 55	Low MPs	96h	No depuration	no	0	X	X	X
4	MP 56	Low MPs	96h	No depuration	no	0	X	X	X
5	MP 57	Low MPs	96h	No depuration	no	0	X	X	X
6	MP 19	Low MPs	96h	No depuration	yes	13	36.18	32.04	6.27
1	MP 22	Low MPs	96h	Presence of food	yes	5	4.12	24.24	16.74
2	MP 23	Low MPs	96h	Presence of food	mortality	X	X	X	X
3	MP 24	Low MPs	96h	Presence of food	yes	1	40.99	40.99	0.00
4	MP 58	Low MPs	96h	Presence of food	no	0	X	X	X
5	MP 59	Low MPs	96h	Presence of food	yes	40	2.71	4.95	1.12
6	MP 60	Low MPs	96h	Presence of food	no	0	X	X	X
1	MP 25	Low MPs	96h	Absence of food	no	0	X	X	X
2	MP 26	Low MPs	96h	Absence of food	yes	1	45.62	45.62	0.00
3	MP 27	Low MPs	96h	Absence of food	yes	1	34.44	34.44	0.00
4	MP 61	Low MPs	96h	Absence of food	no	0	X	X	X
5	MP 62	Low MPs	96h	Absence of food	no	0	X	X	X
6	MP 63	Low MPs	96h	Absence of food	no	0	X	X	X
1	MP 10	High MPs	24h	No depuration	no	0	X	X	X
2	MP 11	High MPs	24h	No depuration	yes	29	43.10	48.62	8.20
3	MP 12	High MPs	24h	No depuration	yes	9	32.42	33.19	4.31
4	MP 46	High MPs	24h	No depuration	yes	12	5.36	12.86	3.47
5	MP 47	High MPs	24h	No depuration	yes	4	16.81	19.69	7.48
6	MP 48	High MPs	24h	No depuration	no	0	X	X	X
1	MP 13	High MPs	24h	Presence of food	no	0	X	X	X
2	MP 14	High MPs	24h	Presence of food	yes	7	33.16	35.50	1.87
3	MP 15	High MPs	24h	Presence of food	yes	14	2.77	9.39	3.83
4	MP 49	High MPs	24h	Presence of food	yes	1	42.96	42.96	0.00
5	MP 50	High MPs	24h	Presence of food	yes	22	2.17	15.16	4.50
6	MP 51	High MPs	24h	Presence of food	yes	3	43.67	47.06	4.97
1	MP 16	High MPs	24h	Absence of food	no	0	X	X	X
2	MP 17	High MPs	24h	Absence of food	yes	3	27.76	29.90	5.44
3	MP 18	High MPs	24h	Absence of food	yes	4	30.93	47.96	20.45
4	MP 52	High MPs	24h	Absence of food	yes	1	42.15	42.15	0.00
5	MP 53	High MPs	24h	Absence of food	no	0	X	X	X
6	MP 54	High MPs	24h	Absence of food	no	0	X	X	X
1	MP 34	High MPs	96h	No depuration	yes	116	38.65	37.62	0.74
2	MP 35	High MPs	96h	No depuration	yes	103	34.04	35.37	2.56
3	MP 36	High MPs	96h	No depuration	yes	24	17.27	25.01	4.50
4	MP 64	High MPs	96h	No depuration	yes	17	35.98	40.98	8.94
5	MP 65	High MPs	96h	No depuration	yes	161	38.74	38.62	2.04
6	MP 66	High MPs	96h	No depuration	mortality	X	X	X	X
1	MP 31	High MPs	96h	Presence of food	yes	2	48.02	48.02	43.68
2	MP 32	High MPs	96h	Presence of food	yes	71	14.95	21.74	2.41
3	MP 33	High MPs	96h	Presence of food	yes	16	54.71	66.11	9.26
4	MP 67	High MPs	96h	Presence of food	Yes	6	34.69	44.89	19.78
5	MP 68	High MPs	96h	Presence of food	no	0	X	X	X
6	MP 69	High MPs	96h	Presence of food	no	0	X	X	X
1	MP 28	High MPs	96h	Absence of food	yes	3	6.38	9.50	5.57
2	MP 29	High MPs	96h	Absence of food	yes	144	31.70	33.50	2.06
3	MP 30	High MPs	96h	Absence of food	yes	139	43.97	48.03	2.07
4	MP 70	High MPs	96h	Absence of food	Yes	18	45.07	49.57	5.64
5	MP 71	High MPs	96h	Absence of food	Yes	2	49.13	49.13	11.08
6	MP 72	High MPs	96h	Absence of food	no	0	X	X	X

Supplementary Table S2. Number of microplastics and their average size in µm (mean ± standard error) according to shape type, *G. duebeni*'s body part and depuration type. Independent replicates were run (R=6) with a total of 96 amphipods tested of which 36 belonged to the control (microplastic free) group. 'Total N of amphipods' in the table show the number of amphipods/body section that had accumulated microspheres and/or fragments.

		NO DEPURATION								
		**Foregut**								
		**Total N of MPs among all amphipods**	**N amphipods with MPs in foreguts**	**Mean MPs/Amph**	**Mean MP size (µm)**	**±SE (µm)**		**Total N of MPs per shape type**		**Total N of MP/foregut**
**Microsphere**	**Spherical**	138	6	23.00	36.50	1.97	**Microsphere**	138	**All MPs**	181
**Fragment**	**Small**	26	6	4.33	8.89	0.18	**Fragment**	43		
	**Flat**	7	1	7.00	68.97	0.05				
	**Cracked**	10	3	3.33	55.17	0.06				
		**Midgut and hindgut**								
		**Total N of MPs among all amphipods**	**N amphipods with MPs in foreguts**	**Mean MPs/Amph**	**Mean MP size (µm)**	**±SE (µm)**		**Total N of MPs per shape type**		**Total N of MP/midgut-hindgut**
**Microsphere**	**Spherical**	107	6	17.83	35.47	1.52	**Microsphere**	107	**All MPs**	308
**Fragment**	**Irregular**	86	8	10.75	15.63	2.19	**Fragment**	201		
	**Flat**	12	3	4.00	104.39	11.42				
	**Cracked**	103	6	17.17	50.21	2.86				
**TOTAL MP/all amphipods**		489								

		DEPURATION IN PRESENCE OF FOOD								
		**Foregut**								
		**Total N of MPs among all amphipods**	**N amphipods with MPs in foreguts**	**Mean MPs/Amph**	**Mean MP size (µm)**	**±SE (µm)**		**Total N of MPs per shape type**		**Total N of MP/foregut**
**Microsphere**	**Spherical**	10	4	2.50	39.12	4.32	**Microsphere**	10	**All MPs**	40
**Fragment**	**Irregular**	25	3	8.33	5.03	2.69	**Fragment**	30		
	**Flat**	0	0	0.00	0.00	0.00				
	**Cracked**	5	1	5.00	26.91	6.12				
		**Midgut and hindgut**								
		**Total N of MPs among all amphipods**	**N amphipods with MPs in foreguts**	**Mean MPs/Amph**	**Mean MP size (µm)**	**±SE (µm)**		**Total N of MPs per shape type**		**Total N of MP/midgut-hindgut**
**Microsphere**	**Spherical**	14	6	2.33	36.31	5.28	**Microsphere**	14	**All MPs**	148
**Fragment**	**Irregular**	66	6	11.00	4.19	2.49	**Fragment**	134		
	**Flat**	43	4	10.75	40.02	4.41				
	**Cracked**	25	8	3.13	42.37	7.13				
**TOTAL MP/all amphipods**		188								

		DEPURATION IN ABSENCE OF FOOD								
		**Foregut**								
		**Total N of MPs among all amphipods**	**N amphipods with MPs in foreguts**	**Mean MPs/Amph**	**Mean MP size (µm)**	**±SE (µm)**		**Total N of MPs per shape type**		**Total N of MP/foregut**
**Microsphere**	**Spherical**	20	6	3.33	38.74	1.69	**Microsphere**	20	**All MPs**	40
**Fragment**	**Irregular**	7	4	1.75	9.13	4.23	**Fragment**	20		
	**Flat**	1	1	1.00	108.90	0.00				
	**Cracked**	12	4	3.00	59.77	8.76				
		**Midgut and hindgut**								
		**Total N of MPs among all amphipods**	**N amphipods with MPs in foreguts**	**Mean MPs/Amph**	**Mean MP size (µm)**	**±SE (µm)**		**Total N of MPs per shape type**		**Total N of MP/midgut-hindgut**
**Microsphere**	**Spherical**	52	6	8.67	34.29	1.71	**Microsphere**	52	**All MPs**	277
**Fragment**	**Irregular**	26	3	8.67	4.73	1.07	**Fragment**	225		
	**Flat**	53	2	26.50	43.56	40.40				
	**Cracked**	146	4	36.50	48.12	5.10				
**TOTAL MP/all amphipods**		317								

Supplementary Table S3. Most common microplastic shape type found in *G. duebeni* body sections according to depuration types. Sizes are given in mean ± SE.

	**Foreguts**			**Midguts-Hindguts**		
	**Shape type**	**Size**	**Total**	**Shape type**	**Size**	**Total**
**No depuration**	Intact spherical	36.50 µm ± 1.97	138	Intact spherical	35.47 µm ± 1.52	107
**Depuration in presence of food**	Small irregular	5.03 µm ± 2.69	25	Small irregular	4.19 µm ± 2.49	66
**Depuration in absence of food**	Intact spherical	38.74 µm ± 1.69	20	Cracked	48.12 µm ± 5.10	146

In the absence of depuration, the most common microplastic shape in *G. duebeni* foregut sections was the intact spherical microplastic type (a total of 138 microplastics of an average size of 36.50 ± 1.97 µm, **Table S3**). After depuration in the presence of food, the most common shape in foreguts was the small irregular microplastic fragment type (25 microplastics of an average size of 5.03 ± 2.69 µm, **Table**

S2). Following depuration in absence of food, the most common shape in foreguts was the whole spherical microplastic (20 microplastics of an average size of 38.74 ± 1.69 µm, **Table S3)**.

Likewise, in the absence of depuration, the most common microplastic shape in midguts-hindguts was the whole spherical microplastic type (a total of 107 microplastics of an average size of 35.47 ± 1.52 µm, **Table S3**) though in very similar numbers to cracked fragments (a total of 103 microplastics of an average size of 50.21 ± 2.86 µm). After depuration in the presence of food, the most common shape in midguts-hindguts were also small irregular fragments (a total of 66) of an average size of 4.19 ± 2.49 µm, **Table S3**). After depuration in the absence of food, the most common shape in midguts-hindguts were cracked fragments (a total of 146) of an average size of 48.12 ± 5.10 µm, **Table S3**).

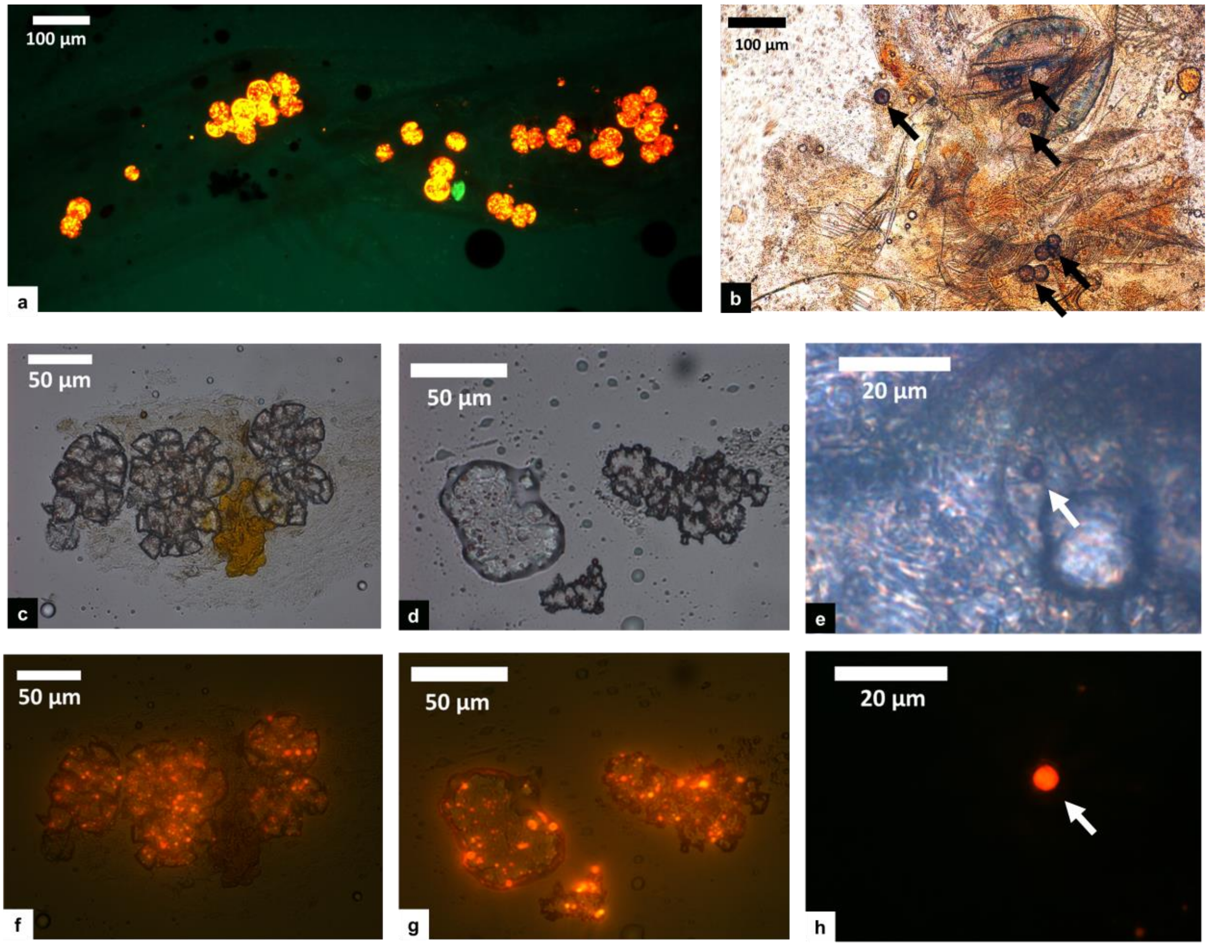

Supplementary Figure S3: Cracked and fragmented plastic particles from *G. duebeni* guts at 10x, 40x or 100x magnification under fluorescence and/or light microscopy; **(a)** microplastics in midgut-hindgut under UV fluorescence at 10x, **(b)** microplastics in foregut under light microscopy at 10x, **(c)** cracked microplastics under light microscopy at 40x, **(d)** flat microplastics under light microscopy at 40x, **(e)** small-irregular microplastics under light microscopy at 100x, **(f)** cracked microplastics under UV fluorescence at 40x, **(g)** flat microplastics under UV fluorescence at 40x and **(h)** small-irregular microplastics under UV fluorescence at 100x.

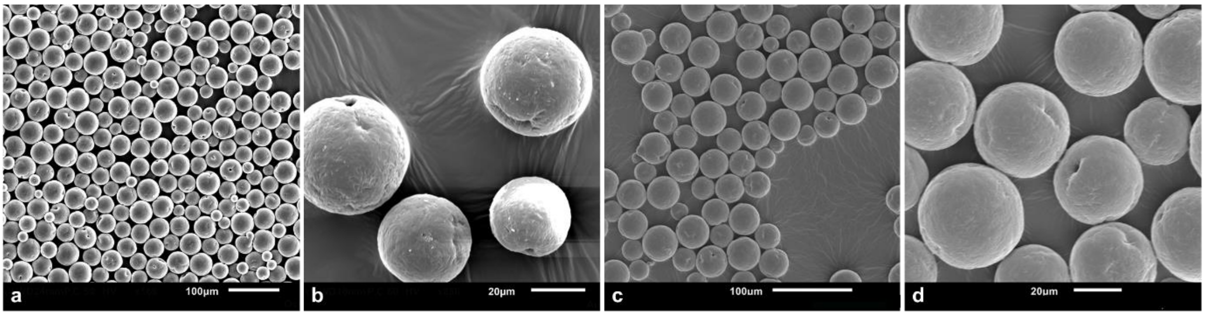

Supplementary Figure S4. Scanning electron microscopy (SEM) images of (**a**) microplastic stock in Tween solution as used in bioassays, (**b**) microplastic stock as dry powder provided by the supplier, (**c**) microplastics frozen at -80ºC for seven days in distilled water and (**d**) microplastics frozen at -80ºC for seven days in Tween solution.

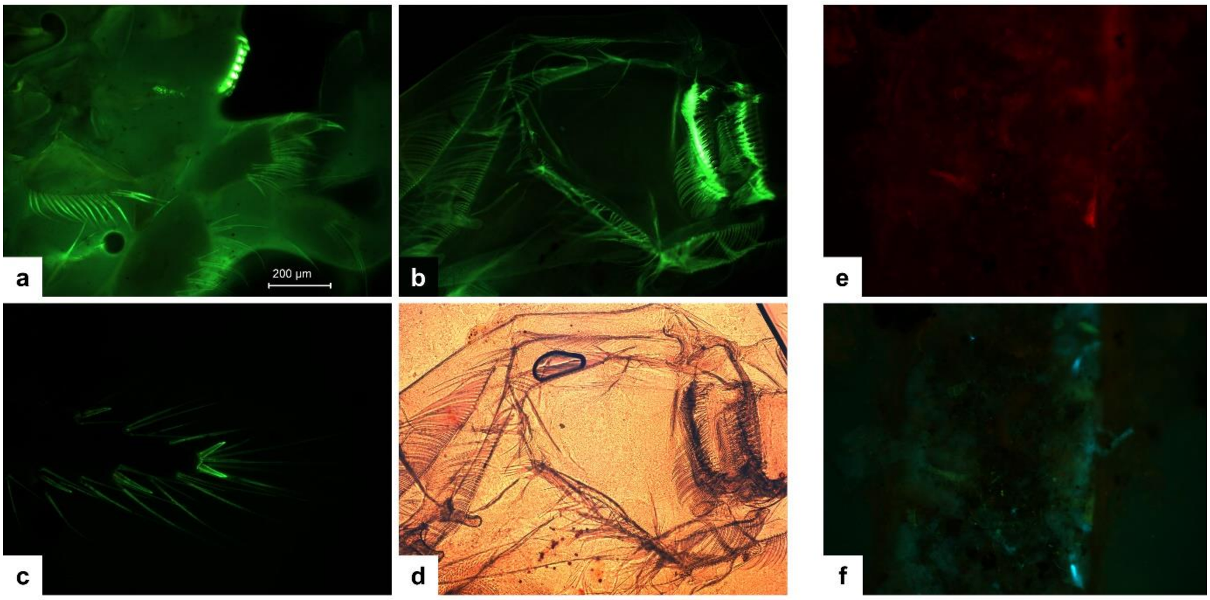

Supplementary Figure S5. Microscope images showing the biological autofluorescence of control *G. duebeni* exoskeleton or mandible tissues (**a,b,c**) under fluorescence green light or brightfield (**d**) and *G. duebeni* gut tissues (**e,f**) showing biological autofluorescence under green light (**e**) or UV light (**f**). All images show tissues from control individuals, not exposed to microplastics or fluorescence dyes.

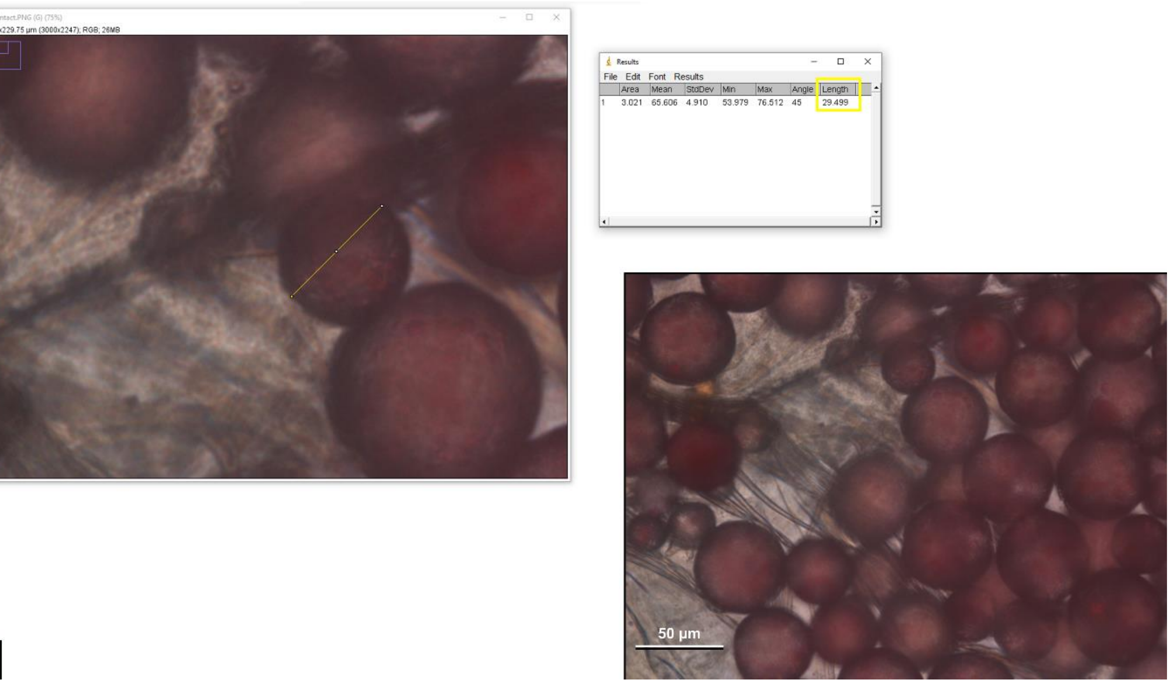
1. intact.PNG (G) (75%)
306.75x229.75 µm (3000x2247); RGB; 26MB
Results
File Edit Font Results
Area Mean StdDev Min Max Angle Length
1 3.021 65.606 4.910 53.979 76.512 45 29.499
50 µm

a

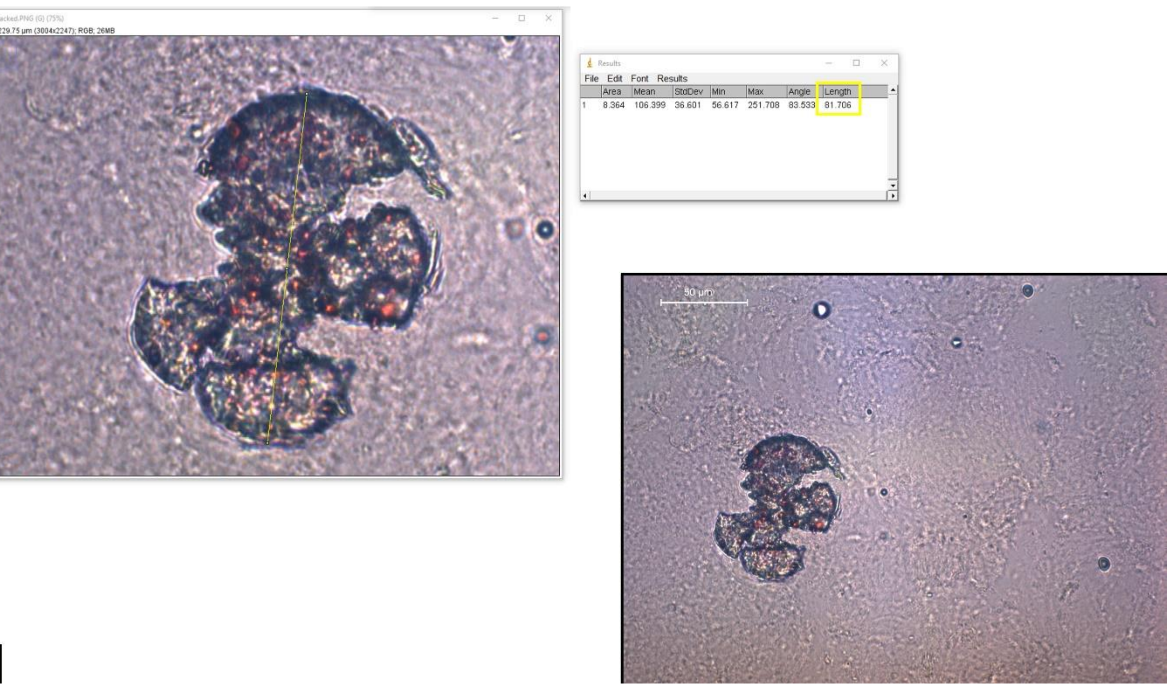
2. cracked.PNG (G) (75%)
307.16x229.75 µm (3004x2247); RGB; 26MB
Results
File Edit Font Results
Area Mean StdDev Min Max Angle Length
1 8.364 106.399 36.601 56.617 251.708 83.533 81.706

b

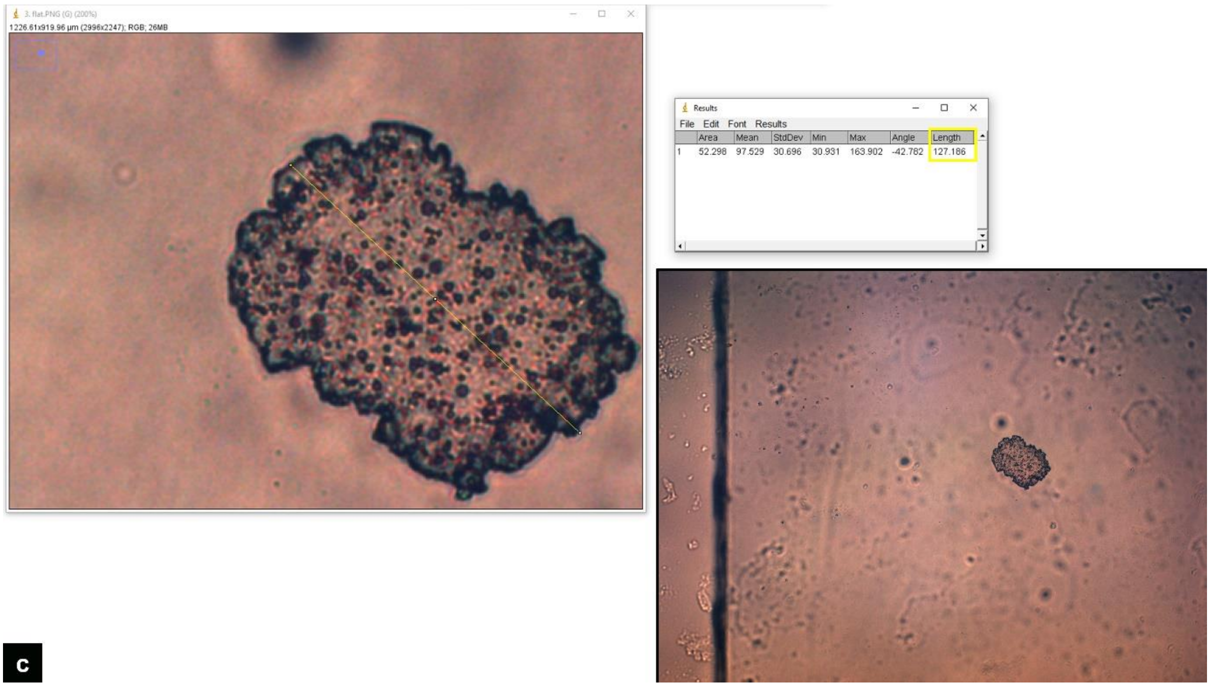

c

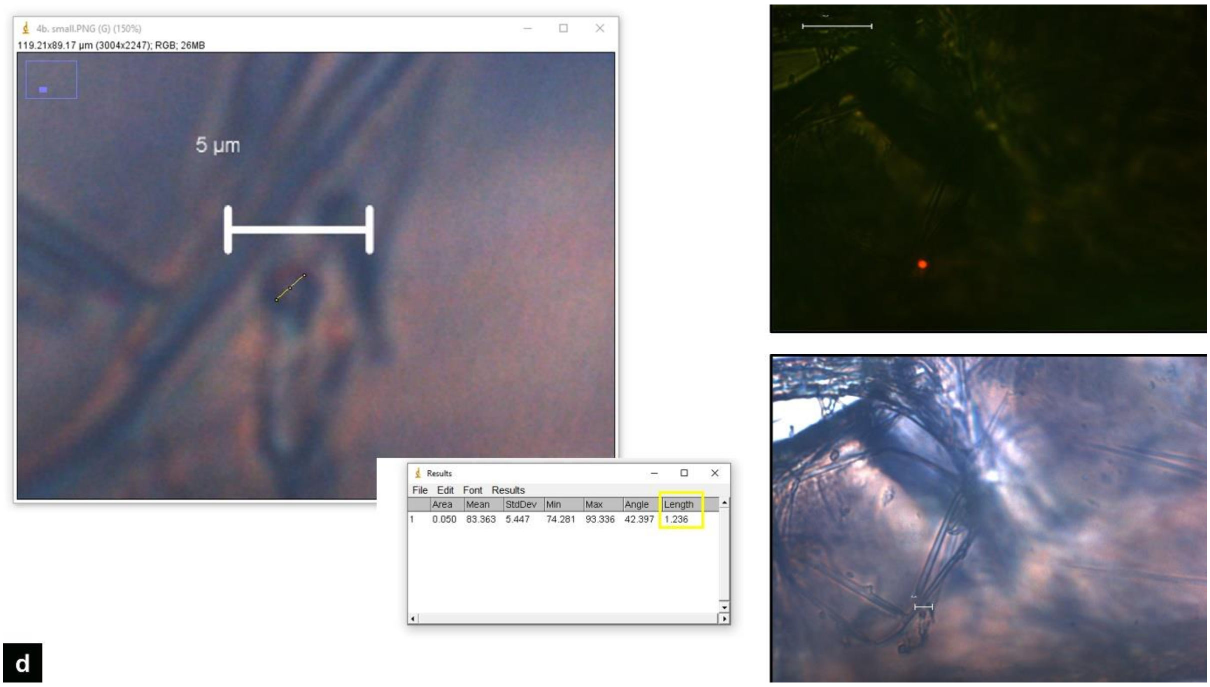

d

Supplementary Figure S6. ImageJ measurements of (**a**) an intact microsphere, (**b**) a cracked fragment, (**c**) a flat fragment and (**d**) a small fragment. Longest length/diameter was measured.
